# A population-based simultaneous fugacity model design for polychlorinated biphenyls (PCBs) transport in an aquatic system

**DOI:** 10.1016/j.mex.2018.07.001

**Published:** 2018-08-09

**Authors:** Xiangfei Sun, Carla A. Ng, Mitchell J. Small

**Affiliations:** a Carnegie Mellon University, Departments of Civil and Environmental Engineering, Pittsburgh, PA 15213, USA; b University of Pittsburgh, Department of Civil and Environmental Engineering, Pittsburgh, PA 15261, USA; c Carnegie Mellon University, Departments of Civil and Environmental Engineering and Engineering and Public Policy, Pittsburgh, PA 15213, USA

**Keywords:** Population-based simultaneous fugacity model, Fugacity capacity variation, Population estimation, Mortality rate, Energy flows, Multicompartment box model

## Abstract

A population-based bioaccumulation fugacity model is designed to simulate the continuous and dynamic transport of polychlorinated bisphenols (PCBs) in an aquatic environment. The extended model is developed based on a previous fugacity model by Campfens and Mackay. The new model identifies each biotic species as a populated compartment and constructs all the exchange routes between organisms and the environment based on known biological processes. The population-based design could assist to uncover the impacts of organism activities on PCB fate and transport in the ecosystem. The new model utilizes the PCB loading as inputs and calculates the PCB distribution in each biotic and environmental compartment simultaneously.

## Highlights

• The extended model adopts a population-based design. Treat each organism as a compartment and estimate the PCB mass based on biota population.
• Establish PCB exchange routes between organisms and the environment, especially the feedback processes from the organism to the environment.
• Predict PCB distribution under both the steady state and the dynamic scenarios.
• Yielding a more realistic simulation among organisms and the environment.

## Graphical abstract

PCBs Exchange between Organism and Environment (New Design).

**Figure:**
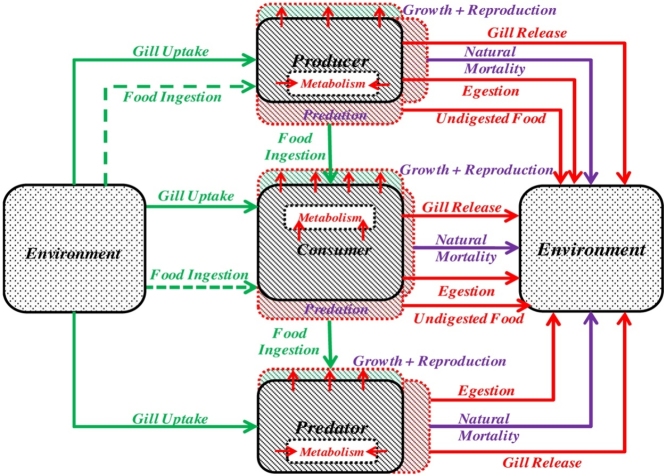

## Specifications table

**Table:** 

Subject area	*Environmental Science*
More specific subject area	*Pollutant Transport*
Method name	*Population-based simultaneous fugacity model*
Name and reference of the original method	*Fugacity-Based Model of PCB Bioaccumulation in Complex Aquatic Food Webs* [1]
Resource availability	*Matlab R2016b, Microsoft Excel 2017*

## Method details

### Model assumptions

1 All compartments are homogeneity and PCBs are evenly distributed inside each compartment. No spreading delay is considered;
2 The biota population size is varied by growth rate, mortality rate, and predation rate;
3 The fugacity capacity varies based on the compartmental temperature. The organism fugacity capacity is also affected by the lipid content. However, we lack proper lipid content variation data. As a compromise, current model assumes constant lipid content for each species.
4 No PCB dechlorination effect and species migration effect is quantitatively considered in this approach.

### General model description

The fundamental approach includes two types of compartments: the environmental compartments (air, water, and sediment) and the biotic compartments. Moreover, the new design adopts the population perspective and estimates pollution exchange rates between the environment and organisms. The accumulated pollutant in each compartment is expressed as [9,17,20]:

(1) $$M_{i}=V_{i}Z_{i}f_{i}$$

Where $M_{i}$ represents the mass of PCBs accumulated in compartment  i; $V_{i}\left(m^{3}\right)$ is the volume of compartment  i; $Z_{i}$ (mol/Pa m^3^) is the fugacity capacity of compartment  i; $f_{i}(Pa)$ is the PCBs fugacity which represents the level of PCBs in compartment  i. Thus, the dynamic change of PCBs in compartment i is estimated as:

(2) $$\frac{dM_{i}}{dt}=\frac{{d(Z}_{i}V_{i}f_{i})}{dt}$$

The formula is transformed through partial difference to become,

(3) $$\frac{df_{i}}{dt}=\frac{1}{Z_{i}V_{i}}\left(\frac{dM_{i}}{dt}-V_{i}f_{i}\frac{{dZ}_{i}}{dt}-Z_{i}f_{i}\frac{{dV}_{i}}{dt}\right)$$

As shown in formula (3), the change of PCBs fugacity in compartment i has three general contributions: the PCBs mass variation ($dM_{i}/dt$), the change in fugacity capacity (${dZ}_{i}/dt$), and the change in compartment volume (${dV}_{i}/dt$). All compartments related to PCBs transport involve at least one of these three general components. To determine the fugacity variation in the certain compartment, we need to separately define the process and parameters in each media according to their physical, chemical, and biological features. Furthermore, we need to define the exchanging terms among different compartments. Moreover, we need to apply a method to estimate the existing biomass/population volume in each biotic compartment for population scale study.

### The fugacity capacity

#### Air

The main components related to PCBs transport in the air are a gaseous phase and aerosol. The fugacity capacity in each component could be expressed as [2]:

(4) $$Z_{1}=\frac{1}{RT_{A}}\;\;\left(Air\right)$$

(5) $$Z_{7}=0.1Z_{1}K_{OA}\;\;\left(Aerosol\right)$$

Where R is the ideal gas constant (8.314 J/mol K); $K_{OA}$ is the octanol-air partition coefficient; $\;T_{A}$ is the air temperature (*K*). Thus, the fugacity capacity in air compartment should be:

(6) $$Z_{A}=Z_{1}+\tau_{1}Z_{7}$$

Where $\tau_{1}$ is the volume fraction of aerosol.

#### Water

The water compartment contains water column and suspended sediment. In some model designs, water compartment also includes aquatic organisms. However, since organisms are isolated and calculated separately, we isolate organisms from the water compartment. The fugacity capacity could be expressed as:

(7) $$Z_{2}=\frac{1}{H}\;\;\left(Water\right)$$

(8) $$Z_{5}=\frac{Z_{2}\rho_{5}\delta_{5}K_{OC}}{1000}\;\left(Suspended\;\;Sediment\right)$$

Where H is the Henry’s Law constant (Pa m^3^/mol); $\rho_{5}$ is the suspended sediment density ($kg/m^{3}$); $\delta_{5}$ is the mass fraction of the organic carbon; $K_{OC}$ is the organic carbon partition coefficient (L/kg), which is approximately 0.41 times of the Kow [3]:

Thus, the capacity of water compartment should be:

(9) $$Z_{W}=Z_{2}+\tau_{2}Z_{5}$$

Where $\tau_{2}$ is the volume fraction of suspended sediment.

According to assumption 1, the water phase and the suspended sediment particle should have identical fugacities during PCB transport. In 2011, LimnoTech provided a study report regarding PCB loading patterns in Lake Ontario [4]. According to the study, the PCB input of Lake Ontario in 2005 came from air transmission (20%) and water flows (80%). Moreover, a detailed analysis on aquatic PCBs input indicates a 70%/30% allocation between dissolved PCBs (water column) and particle PCBs (suspended sediment). Thus,

(10) $$\begin{array}{c} \frac{m_{water}^{PCBs}}{m_{susp.\;sedi.}^{PCBs}}=\frac{0.56}{0.24}=\frac{V_{water}Z_{water}f_{water}}{V_{susp.\;sedi.}Z_{susp.\;sedi.}f_{susp.\;sedi.}}\\ \frac{V_{water}Z_{2}f_{water}}{\tau_{2}V_{water}Z_{5}f_{susp.\;sedi.}}=\frac{1000V_{water}Z_{2}}{\tau_{2}V_{water}Z_{2}\rho_{5}\delta_{5}K_{OC}}=\frac{0.56}{0.24}\\ \frac{1000}{\tau_{2}\rho_{5}\delta_{5}K_{OC}}=\frac{0.56}{0.24} \end{array}$$

Thus

(11) $$\tau_{2}=\frac{3000}{7\rho_{5}\delta_{5}K_{OC}}$$

#### Sediment

The vertical homogeneity conversion of the sediment compartment is difficult since the PCBs content with sediment depth depends on the PCB contamination level during the deposition period. As a temporary compromise, the current sediment compartment only includes the very top layer of bio-active sediment (≈0.1 m) which contains 10% dry residual mixed with around 90% of the saturated water (in volume fraction). The sediment is considered as flooded sediment, where little air existed in the compartment [19]. As a result, the sediment compartment is a mixture of water and sediment solid with organic particle attached to the organic matters. The solid sediment particle phase has a fugacity capacity as:

(12) $$Z_{4}=\frac{Z_{2}\rho_{4}\delta_{4}K_{OC}}{1000}\;\left(dry\;\;sediment\right)$$

Thus, the fugacity capacity of the sediment compartment is:

(13) $$Z_{S}=\left(1-\tau_{3}\right)Z_{2}+\tau_{3}Z_{4}$$

Where $\tau_{3}$ is the volume fraction of solid sediment.

For accurate estimation, the fraction of organic carbon in flooded sediment could be calculated through water content and dry bulk density [5]:

(14) $$Dry\;\;Bulk\;\;Density\;\left(\text{g}/\text{c}{\text{m}}^{3}\right)=1.776-0.363lnOC$$

Where OC is the organic carbon concentration (mg/dw g). The inorganic sediment particle density is conventionally taken 2.65 g/cm^3^; the density of organic matters can be corrected assuming a density of 1.25 g/cm^3^. Thus, the sediment solid density can be expressed as:

$$Soild\;\;Density\;\left(\text{g}/\text{c}{\text{m}}^{3}\right)=1.25*\left(\%\;OM\right)+2.65*\left(1-\%\;OM\right)$$

(15) OM=1.7OC

Thus, the water content is:

(16) $$\tau_{3}=\left(1-\frac{Dry\;Bulk\;Density}{Soild\;\;Density}\right)\times 100\%$$

Finally, the fraction of OC is:

(17) $$\frac{1.776-0.363ln(1000*OM/1.7)}{2.65-1.4\%\;OM}=1-\tau_{3}$$

This equation means we can use water content to estimate the fraction of organic carbon in the sediment.

#### Organism

According to Mackay, the fugacity capacity of biota is defined as [2]:

(18) $$Z_{B}=LZ_{L}=LZ_{O}=LZ_{W}K_{OW}$$

Where L is the lipid fraction in the organism.

### PCB mass variation

The PCB mass variation, or $dM_{i}/dt$, is defined as the PCB mass enters or exits the system with general transport processes. The general form for the changes of fugacity in compartment  i can be expressed as,

(19) $$\frac{dM_{i}}{dt}=E_{i}+\sum_{j=1}^{n}\left(D_{ji}f_{j}\right)-D_{T_{i}}f_{i}$$

In this formula, i represents the different media; j represents other media that interact with media $i;M_{i}\left(mol\right)$ represents the current PCB mass in medium i; t(day) represents the PCB transport and allocation time; $\;E_{i}(mol/day)$ represents the direct pollution exchange rate to medium  i; $f_{j}(Pa)$ represents the PCB fugacity in medium  j; $f_{i}\left(Pa\right)$ represents the fugacity of medium  i; $D_{ji}$ (mol/Pa day) represents the PCB transport processes from medium j to medium  i (i≠j); $D_{T_{i}}$ (mol/Pa day) represents the total PCB elimination/exit from medium  i.

#### Air

Pollutant transport processes related to air compartment include three processes: the inter-media exchange, the self-elimination, the systematic exchange [6]. During the inter-media exchange, the entrée is mainly through water volatilization (air-water diffusion,${\;D}_{V}$), while the exit pathways include absorption (water-air diffusion,${\;D}_{V}$), wet dissolution ($D_{RWW}$), dry deposition ($D_{QDW}$), wet particle deposition ($D_{QWW}$). Since no biota is considered in the air, no direct exchange between the air compartment and any organisms. The self-elimination, or reaction ($R_{A}$) within the compartment generally eliminate contaminate through photodegradation and is related to the compartmental-based lifetime. Finally, the systematic exchange is mainly through the advections ($D_{AI}/D_{AO}$). As a result, the fugacity variation in the air compartment could be written as:

(20) $$\frac{dM_{A}}{dt}=\left({f_{in1}D}_{AI}-{\;f_{A}D}_{AO}\right)+{\;D}_{V}\left(f_{W}-f_{A}\right)-(D_{RWW}+D_{QDW}+D_{QWW}+R_{A})f_{A}$$

Where

(21) $$\text{Diffusion}:\;\;D_{V}={\left(\frac{1}{k_{VA}A_{AW}Z_{1}}+\frac{1}{k_{VW}A_{AW}Z_{2}}\right)}^{-1}$$

(22) $$\text{Wet}\;\;\text{Dissolution}:\;\;D_{RWW}=A_{AW}U_{Q}Z_{2}$$

(23) $$\text{Dry}\;\;\text{Deposition}:\;\;D_{QDW}=A_{AW}U_{Q}v_{Q}Z_{7}$$

(24) $$\text{Wet}\;\text{Particle}\;\;\text{Deposition}:\;\;D_{QWW}=A_{AW}U_{R}Qv_{Q}Z_{7}$$

(25) $$\text{Reaction}:\;\;R_{A}=\frac{V_{A}Z_{A}}{t_{A}}$$

(26) $$\text{Advection}\;\;\text{Input}:\;\;D_{AI}=G_{in}Z_{A}$$

(27) $$\text{Advection}\;\;\text{Output}:\;\;D_{AO}=G_{out}Z_{A}$$

Thus,

(28) $$Z_{A}V_{A}\frac{df_{A}}{dt}=\left({f_{in1}G}_{in}-{f_{A}G}_{out}\right)Z_{A}+{\left(\frac{1}{k_{VA}A_{AW}Z_{1}}+\frac{1}{k_{VW}A_{AW}Z_{2}}\right)}^{-1}\left(f_{W}-f_{A}\right)-(A_{AW}U_{Q}Z_{2}+A_{AW}U_{Q}v_{Q}Z_{7}+A_{AW}U_{R}Qv_{Q}Z_{7}+\frac{V_{A}Z_{A}}{t_{A}})f_{A}$$

#### Biota

The organisms are discussed previously for better understanding their interactions with the environment phases. In this study, the definition of the inter-exchange process among different biota groups occurs only within the food web, while the processes with the environmental groups are identified as a systematic exchange. In the inter-exchange process, PCBs are absorbed by organisms through food ingestion ($D_{FI}$) and are released through predation ($D_{Pred}$).

When studying the PCB transport between environment and organism in water compartment, organisms are divided into pelagic and benthic species, because habitat location will lead to different calculation method PCB exchange rate. Gill uptake is one of the primary routes to transfer PCBs into organisms ($D_{GGW}/D_{GGS}$) [18]. The pathways to transport PCBs to the environment includes gill release ($D_{GLW}/D_{GLS}$), natural mortality ($D_{MD}$), and egestion ($D_{E}$). Egestion is combined by the undigested food ($1-E_{D}$) and PCB exchange between the gut and the feces ($D_{Ethe\;X}$). Undigested food is usually estimated as a proportion of the total food ingestion, while the gut/feces exchange rate is estimated through trophic magnification factor (TMF) and trophic levels [16]. The PCB self-elimination in biota group is mainly through metabolism ($R_{B}$).

Considering the existence of the decomposing process, we assume that the PCB inside dead organisms caused by natural mortality will be initially decomposed and released to the environment before regaining through the food web. For the pelagic species, PCBs from decomposed organisms return to both water and sediment; for benthic groups, all released PCBs go to the sediment compartment. Thus, the changes of fugacity in biota could be expressed as:

*Pelagic species*

(29) $$\frac{dM_{P}}{dt}=D_{GG}f_{W}+\sum_{i=pelagic\&i\neq j}^{n}p_{ij}D_{FIj}f_{j}-D_{GLW}f_{i}-\left(R_{Bi}+D_{MDi}+D_{Predi}\right)f_{i}-\sum D_{EXi}f_{j}$$

*Benthic species*

(30) $$\frac{dM_{B}}{dt}=D_{GG}f_{S}+\sum_{i=benthic\&i\neq j}^{n}p_{ij}D_{FIj}f_{j}-D_{GLS}f_{i}-\left(R_{Bi}+D_{MDi}+D_{Predi}\right)f_{i}-\sum D_{EXi}f_{j}$$

Where

(31) $$Gill\;\;Uptake:\;D_{GG}=k_{1}V_{P}\rho_{B}Z_{W}$$

(32) $$Food\;\;Ingestion:\;D_{FIi}=E_{D}\frac{\rho_{i}V_{Pi}G_{Di}Z_{Bj}}{W_{Bi}}$$

(33) $$Gill\;\;Release:\;D_{GL}=D_{GG}$$

(34) $$Metabiolism:\;R_{Bi}=V_{P}Z_{i}k_{M}$$

#### PCB exchange between the gut and the feces

The PCB exchange rate between the gut and the feces can be calculated through TMF and trophic levels. TMF, or trophic magnification factor, could be used to evaluate the proportion of PCB escape from the system through feces [16]. The species trophic level can be calculated by the following formula [7]:

(35) $${TL}_{i}=1+\sum {TL}_{j}∙p_{ij}$$

Where ${TL}_{j}$ represents the fractional trophic level of prey *j*, and $p_{ij}$ represents the fraction of *j* in the diet of *i*. The PCB released through the fence is then decided by the true TMF differences between food and diet:

(36) $$D_{EX}=\frac{E_{D}{\rho_{i}V}_{Pi}G_{Di}}{W_{Bi}}\sum_{i\neq j}^{n}\frac{p_{ij}Z_{Bj}f_{j}}{\left({TL}_{i}-{TL}_{j}\right)}*TMF$$

##### Predation

(37) $$D_{Predi}=\sum_{i\neq k}^{n}\frac{{\rho_{k}V}_{Pk}p_{ik}G_{Dk}}{W_{Bk}}=\sum_{i\neq k}^{n}\frac{{0.022e^{0.06T}\rho_{k}V}_{Pk}p_{ik}}{W_{Bk}^{0.15}}*Z_{Bi}$$

#### Natural mortality (mortality without predation)

The natural mortality rate is estimated by Then et al. in 2015, who used over 200 fish species to evaluate the current existing empirical models for natural mortality rate estimation [8]. We selected one of the best models as a basis for estimating the natural mortality loss.

(38) $$D_{MDi}=\frac{4.899t_{max}^{-0.916}V_{Pi}}{365000}*Z_{Bi}$$

Where, $t_{max}$ is the maximum surviving time for species  i (years);

#### Growth dilution

According to the new representation in formula (1) through (3), the growth dilution does not belong to the first category, $\frac{dM_{i}}{dt}$, since there is no actual entrée or exit of any PCB during the process. It is merely a volume change. As a result, it should be moved to the third part.

In sum, the extended expressions for formula (29) and (30) are:

*Pelagic species*

(39) $$\frac{dM_{BP}}{dt}=k_{1i}V_{Pi}\rho_{B}Z_{W}f_{W}+\frac{E_{D}{\rho_{i}V}_{Pi}G_{Di}}{W_{Bi}}\sum_{i\neq j}^{n}\frac{{TL}_{i}-{TL}_{j}-1}{{TL}_{i}-{TL}_{j}}*TMF*p_{ij}Z_{Bj}f_{j}-f_{i}\left(k_{1i}V_{Pi}\rho_{B}Z_{W}+V_{Pi}Z_{i}k_{Mi}+\frac{4.899t_{max}^{-0.916}V_{Pi}}{365000}*Z_{Bi}+\sum_{i\neq k}^{n}\frac{{0.022e^{0.06T}\rho_{k}V}_{Pk}p_{ik}}{W_{Bk}^{0.15}}*Z_{Bi}\right)$$

*Benthic species*

(40) $$\frac{dM_{BB}}{dt}=k_{1i}V_{Pi}\rho_{B}Z_{W}f_{S}+\frac{E_{D}{\rho_{i}V}_{Pi}G_{Di}}{W_{Bi}}\sum_{i\neq j}^{n}\frac{{TL}_{i}-{TL}_{j}-1}{{TL}_{i}-{TL}_{j}}*TMF*p_{ij}Z_{Bj}f_{j}-f_{i}\left(k_{1i}V_{Pi}\rho_{B}Z_{W}+V_{Pi}Z_{i}k_{Mi}+\frac{4.899t_{max}^{-0.916}V_{Pi}}{365000}*Z_{Bi}+\sum_{i\neq k}^{n}\frac{{0.022e^{0.06T}\rho_{k}V}_{Pk}p_{ik}}{W_{Bk}^{0.15}}*Z_{Bi}\right)$$

#### Water

The pollution transport through water is more complex than the air section because of the existence of organisms. To achieve fidelity, PCB exchange processes between the environment and organisms have been built. Similarly, the pollutant exchange in the water section is divided into three parts. The entrée processes in intermedia exchange include

I Air-Water: absorption (water-air diffusion,${\;D}_{V}$), wet dissolution ($D_{RWW}$), dry deposition ($D_{QDW}$), wet particle deposition ($D_{QWW}$);
II Water-Sediment: diffusion ($D_{Y}$), deposition ($D_{DS}$);
III Water-Biota: gill release ($D_{GLW}$), death loss (mortality, $D_{ML}$), egestion ($Q_{E}$);

The exit processes in intermedia exchange:

I Air-Water: volatilization (air-water diffusion,${\;D}_{V}$);
II Water-Sediment: diffusion ($D_{Y}$), resuspension ($D_{RS}$);
III Water-Biota: gill uptake ($D_{GGW}$);

The self-elimination, or reaction ($R_{W}$) within the compartment generally eliminate contaminate under a first-order decay rate, which is relative to its compartment-based lifetime. Finally, the systematic exchange is mainly through the advections in/out ($D_{WI}/D_{WO}$) of the system. As a result, the fugacity variation in the water compartment could be written as:

(41) $$\frac{dM_{W}}{dt}=\left({f_{in2}D}_{WI}-{f_{W}D}_{WO}\right)+D_{V}\left(f_{A}-f_{W}\right)+\left(D_{RWW}+D_{QDW}+D_{QWW}\right)f_{A}+\sum_{i=pelagic}^{n}\left[{D_{GLi}f_{i}-D}_{GGi}f_{W}\right]{-{\;R}_{W}f_{W}\;+\;D}_{Y}\left(f_{S}-f_{W}\right)+D_{RS}f_{S}-D_{DS}f_{W}$$

(42) $$Advection\;in/out:\;\;D_{WI}=D_{WO}=G_{W}Z_{W}$$

(43) $$Water-sediment\;\;Diffusion:\;\;D_{Y}=\frac{K_{SW}}{Y_{4}}A_{SW}Z_{2}$$

(44) $$Water-Sediment\;\;Deposition:\;D_{DS}=U_{DP}A_{SW}Z_{5}$$

(45) $$Water-Sediment\;\;Resuspension:\;D_{RS}=U_{RS}A_{SW}Z_{4}$$

(46) $$Reaction:\;R_{W}=\frac{V_{W}Z_{W}}{t_{W}}$$

In sum the extended expression for water compartment could be written as:

(47) $$\frac{dM_{W}}{dt}=G_{W}Z_{W}\left(f_{in2}-f_{W}\right)+{\left(\frac{1}{k_{VA}A_{AW}Z_{1}}+\frac{1}{k_{VW}A_{AW}Z_{2}}\right)}^{-1}\left(f_{A}-f_{W}\right)+\left(A_{AW}U_{Q}Z_{2}+A_{AW}U_{Q}v_{Q}Z_{7}+A_{AW}U_{R}Qv_{Q}Z_{7}\right)f_{A}\;+\;\frac{B_{MS}}{Y_{4}}A_{SW}Z_{2}\left(f_{S}-f_{W}\right)+U_{RS}A_{SW}Z_{4}f_{S}-U_{DP}A_{SW}Z_{5}f_{W}-\frac{V_{W}Z_{W}f_{W}}{t_{W}}+\sum_{i=pelagic}^{n}\left[\left(f_{i}-f_{W}\right)k_{1i}V_{Pi}\rho_{B}Z_{W}\right]$$

#### Sediment

Similarly, as water compartment, the sediment compartment includes biotic activities. Thus it also contains similar processes. The entrée processes in intermedia exchange include

I Water-Sediment: diffusion ($D_{Y}$), resuspension ($D_{RS}$);
II Sediment-Biota: gill release ($D_{GL}$), egestion ($Q_{E}$);
III Water-Biota: part of the egestion ($Q_{E}$);

The exit processes in intermedia exchange:

I Water-Sediment: diffusion ($D_{Y}$), deposition ($D_{DS}$);
II Sediment-Biota: gill uptake ($D_{GG}$);

The self-elimination, or reaction ($R_{W}$) within the compartment eliminates PCB through biodegradation (aerobic remediation only), which is relative to its compartment-based lifetime. The sediment compartment does not have a direct PCB input route, but sediment compartment can push PCBs out of the system by deposition. As a result, the fugacity variation in the sediment compartment could be written as:

(48) $$\frac{dM_{S}}{dt}=\sum_{i=1}^{n}\frac{E_{D}{\rho_{i}V}_{Pi}G_{Di}}{W_{Bi}}\sum_{i\neq j}^{n}\frac{TMF*p_{ij}Z_{Bj}f_{j}}{{TL}_{i}-{TL}_{j}}+\sum_{i=1}^{n}\left(1-E_{D}\right)*\frac{{\rho_{i}V}_{Pi}G_{Di}}{W_{Bi}}\sum_{i\neq j}^{n}p_{ij}Z_{Bj}f_{j}+\sum_{i=1}^{n}D_{MDi}f_{i}+\sum_{i=benthic}^{n}\left[{D_{GLi}f_{i}-D}_{GGi}f_{S}\right]{+D_{DS}f_{W}-{\;R}_{S}f_{S}-\;D}_{Y}\left(f_{S}-f_{W}\right)-D_{RS}f_{S}$$

(49) $$Deposition:\;D_{SO}=G_{S}Z_{S}$$

(50) $$Reaction:\;R_{S}=V_{S}Z_{S}/t_{S}$$

In sum the extended expression for sediment compartment could be written as:

(51) $$\frac{dM_{S}}{dt}=\sum_{i=j}^{n}\frac{E_{D}{\rho_{i}V}_{Pi}G_{Di}}{W_{Bi}}\sum_{i\neq j}^{n}\frac{TMF*p_{ij}Z_{Bj}f_{j}}{{TL}_{i}-{TL}_{j}}+\sum_{i=j}^{n}\left(1-E_{D}\right)*\frac{{\rho_{i}V}_{Pi}G_{Di}}{W_{Bi}}\sum_{i\neq j}^{n}p_{ij}Z_{Bj}f_{j}+\sum_{i=benthic}^{n}\left[\left(f_{i}-f_{S}\right)k_{1i}V_{Pi}\rho_{B}Z_{W}\right]+U_{DP}A_{SW}Z_{5}f_{W}-\frac{V_{S}Z_{S}f_{S}}{t_{S}}-\;\frac{B_{MS}}{Y_{4}}A_{SW}Z_{2}\left(f_{S}-f_{W}\right)-U_{RS}A_{SW}Z_{4}f_{S}+\sum_{i=1}^{n}\frac{4.899t_{max}^{-0.916}V_{Pi}}{365000}*Z_{Bi}f_{i}$$

### Fugacity capacity variation

#### Air

The fugacity capacity of air compartment could be expressed as:

(52) $$Z_{A}=\;\frac{1}{RT_{A}}\;\left(\tau_{1}0.1K_{OA}+1\right)$$

According to Li et al., the Octanol/Air partition coefficient is temperature sensitive with an estimation of:

(53) $$logK_{OA}\left(T\right)=\frac{a}{T_{A}}+b$$

Thus, the fugacity capacity variation in air compartment is expressed as:

(54) $$\frac{{dZ}_{A}}{dt}=\frac{d(\frac{\tau_{1}0.1K_{OA}+1}{RT_{A}}\;)}{dt}=-\frac{\;\left(\tau_{1}0.1K_{OA}+1\right)}{RT_{A}^{2}}\frac{dT_{A}}{dt}+\frac{\tau_{1}0.1}{RT_{A}}\frac{d(K_{OA})}{dt}$$

For

$$\frac{d(K_{OA})}{dt}=\frac{d(10^{\frac{a}{T_{A}}+b})}{dt}=-\frac{ln(10)10^{\frac{a}{T_{A}}+b}}{T_{A}^{2}}\frac{dT_{A}}{dt}$$

Thus,

(55) $$\frac{{dZ}_{A}}{dt}=\frac{d(\frac{\tau_{1}0.1K_{OA}+1}{RT_{A}}\;)}{dt}=-\left[\frac{\;\left(\tau_{1}0.1K_{OA}+1\right)}{RT_{A}^{2}}+\frac{\tau_{1}0.1ln(10)10^{\frac{a}{T_{A}}+b}}{RT_{A}^{3}}\right]\frac{dT_{A}}{dt}$$

#### Water

The fugacity capacity in water could be expressed as:

(56) $$Z_{W}=\frac{1}{H}\left(1+0.41*\frac{{\tau_{2}\rho }_{5}\delta_{5}K_{OW}}{1000}\right)$$

Where H is Henry’s law constant. According to research by Schwarzenbach in 2002, the Henry’s Law constant could be affected by the temperature with the following formula, also as known as van’t Hoff correction [9,10]:

(57) $$H\left(T_{W}\right)=H^{ref}exp\left[-\frac{\Delta U_{AW}}{R}\left(\frac{1}{T_{W}}-\frac{1}{T^{ref}}\right)\right]$$

Where $\;H^{ref}$ is the referenced Henry’s Law constant at $T^{ref}$; $U_{AW}$ is the is the difference in internal energies of PCB in the phase change from air to water (kJ/mol). Similarly, the $K_{OW}$ also adept in van’t Hoff correction:

(58) $$K_{OW}\left(T\right)=K_{OW}^{ref}exp\left[-\frac{\Delta U_{OW}}{R}\left(\frac{1}{T}-\frac{1}{T^{ref}}\right)\right]$$

Where $\Delta U_{OW}$ is the internal energies requirement for PCB going from octanol to water (kJ/mol). However, the $K_{OW}$is much less sensitive to the temperature variation. In this study, we can assume a constant $K_{OW}$ to simplify the calculation. Thus, the fugacity rate of change in water is:

(59) $$\frac{{dZ}_{W}}{dt}=\frac{d\left(\frac{1+0.41*\frac{{\tau_{2}\rho }_{5}\delta_{5}K_{OW}}{1000}}{H}\right)}{dt}=\frac{1}{H^{2}}\frac{dH}{dt}$$

And

(60) $$\frac{dH}{dt}=\frac{d\left\{H^{ref}exp\left[-\frac{\Delta U_{AW}}{R}\left(\frac{1}{T_{W}}-\frac{1}{T^{ref}}\right)\right]\right\}}{dt}=\frac{H*\Delta U_{AW}}{RT_{W}^{2}}\frac{dT_{W}}{dt}$$

Finally,

(61) $$\frac{{dZ}_{W}}{dt}=-\left(1+0.41*\frac{{\tau_{2}\rho }_{5}\delta_{5}K_{OW}}{1000}\right)\frac{\Delta U_{AW}}{HRT_{W}^{2}}\frac{dT_{W}}{dt}$$

#### Sediment

The fugacity capacity in sediment could be expressed as:

(62) $$Z_{S}=\left(1-\tau_{3}\right)Z_{2}+\tau_{3}Z_{4}=\left(1-\tau_{3}\right)Z_{2}+\tau_{3}\frac{Z_{2}\rho_{4}\delta_{4}K_{OC}}{1000}$$

Thus,

(63) $$Z_{S}=\frac{1-\tau_{3}}{H}+\frac{{0.41\tau }_{3}\rho_{4}\delta_{4}K_{OW}}{1000H}$$

Where $\rho_{4}$ is the sediment density (kg/L). Thus the fugacity capacity change in sediment is:

(64) $$\frac{{dZ}_{S}}{dt}=\frac{d(\frac{1-\tau_{3}}{H}+\frac{{0.41\tau }_{3}\rho_{4}\delta_{4}K_{OW}}{1000H})}{dt}=\left(1-\tau_{3}+\frac{{0.41\tau }_{3}\rho_{4}\delta_{4}K_{OW}}{1000}\right)\frac{1}{H^{2}}\frac{dH}{dt}$$

Finally,

(65) $$\frac{{dZ}_{S}}{dt}=\left(1-\tau_{3}+\frac{{0.41\tau }_{3}\rho_{4}\delta_{4}K_{OW}}{1000}\right)\frac{\Delta U_{AW}}{HRT_{W}^{2}}\frac{dT_{W}}{dt}$$

#### Biota

According to Mackay, the fugacity capacity of biota is defined as:

(66) $$Z_{B}=LZ_{L}=LZ_{O}=LZ_{4}K_{OW}$$

Where L is the lipid fraction in biota, then

(67) $$\frac{{dZ}_{B}}{dt}=\frac{d\left(LZ_{4}K_{OW}\right)}{dt}=Z_{W}K_{OW}\frac{dL}{dt}+LK_{OW}\frac{dZ_{W}}{dt}$$

Then

(68) $$\frac{{dZ}_{B}}{dt}=Z_{W}K_{OW}\frac{dL}{dt}-LK_{OW}\frac{\Delta U_{AW}}{HRT_{W}^{2}}\frac{dT_{W}}{dt}$$

### Compartment volume variation

#### Environmental compartment

In this study, we assume no volume change occurs in the environmental compartment.

#### Organism volume estimation through natural mortality, growth rate, and predation

The organism population size is another essential factor for the new model. Pre-existing methods for biomass size estimation involve field investigation and measurement. To improve the estimation of biomass volume, we develop an energy-mass method, which could estimate the primary producers’ biomass in most trophic structures. In this method, we use energy flow to estimate the biomass of the primary producers and mass balance to estimate all following species.

The estimation begins with primary producers. To begin with, two assumptions must be considered. First, it is assumed that the plant cover, such as grassland, forest, wetland, and so on, has long been existing and stabilized before the estimation. Second, solar energy is identified as the primary energy source in the ecosystem. Solar energy input is the fundamental source to determine the scale of the primary producer. However, it is not the only dominant factor to control the plant biomass. The amount of the plant biomass is also determined by the nutritional condition, water supply, and for aquatic ecosystems, light penetration. In general, most of these parameters cannot be measured directly, but they can be estimated via other parameters, such as photosynthetic efficiency and vegetation coverage [11]. Based on the pre-assumptions, the following formula is used for plant scale estimation:

(69) $$m_{phytoplankton,\;algae}=\frac{E_{Solar}\times \varphi_{i}^{PE}\times \sigma_{i}^{C}\;\times \varphi_{i}^{T}\times \vartheta_{i}^{C}\times A}{\varphi_{i}^{C}}\times \tau_{i}$$

Where $E_{Solar}$ (J/s m^2^) represents the total solar energy input to the unit surface; $\varphi_{i}^{PE}\left(\%\right)$ represents the photosynthetic efficiency of plant i; $\sigma_{i}^{C}\left(\%\right)$ represents vegetation coverage rate of each type of plant in the study area; $\varphi_{i}^{T}\left(\%\right)$ is the energy transport factor, the efficient proportion of energy stored in the system; $\vartheta_{i}^{C}(g/J)$ represents the carbon production factor which is the energy transferred to carbon in the system; $\varphi_{i}^{C}(\%)$ represents the carbon fraction, that is, the weight percentage of carbon in the target organism i; $\tau_{i}\left(days\right)$ represents the average lifetime of the species; *A*(m^2^) is the surface area.

In formula (69), the total solar radiation is acquired from the Solargis [12]. Notice that in the current study, the Photosynthetic Efficiency and Carbon Production Factor are usually measured together. The combined parameter of Photosynthetic Efficiency and Carbon Production Factor is based on the Green Solar Collector; converting sunlight into algal biomass [13]. The estimation of the biomass growth efficiency not only involves the photosynthesis efficiency but also takes into account daily consumption for organism growth and self-maintenance. The vegetation coverage rate can also be found in books [14] and the USGS GAP Land Cover Data Set. The calculation results are expressed as volume or mass since the density of most aquatic organisms is near water density. According to the observation data in Lake Ontario [15], the biomass density of phytoplankton is around 0.01–1 g/m^3^. The formula (69) calculation results, depending on the coverage rate and seasonal features, are around 0.03–1.5 g/m^3^.

The next step is to calculate the higher trophic level biomass. Since all consumers gain their energy through food ingestion, it is continent to use food mass flows to monitor the biomass in each species. In current model design, we assume constant population sizes among all the biotic compartments in the ecosystem. The population size could increase through growth/reproduction and lose its size through natural mortality and predation. We do not consider disasters or incidents which could dramatically alter the population scale. Thus, the food mass flow could be written as:

(70) G=P+M

Where G represents growth rate (${day}^{-1}$) [18], P represent predation rate (${day}^{-1}$), and M is the mortality rate (${day}^{-1}$).

The expressions for growth rate, predation rate, and natural mortality rate are:

(71) $$Growth\;\;Rate:\;G=k_{Gi}V_{Pi}=0.00586\;{\left(1.113\right)}^{T-20}\;{\left(1000W_{Bi}\right)}^{-0.2}\;V_{Pi}$$

(72) $$Predation\;\;Rate:\;P=\sum_{i\neq k}^{n}\frac{0.022e^{0.06T}\rho_{k}V_{Pk}p_{ik}}{W_{Bk}^{0.15}}$$

(73) $$Natrual\;\;Mortality\;\;Rate:\;M=\frac{4.899t_{\text{max}}^{-0.916}V_{Pi}}{365000}$$

As a result,

(74) $$\frac{dV_{i}}{dt}=0.00586\;{\left(1.113\right)}^{T-20}\;{\left(1000W_{Bi}\right)}^{-0.2}\;V_{Pi}-\frac{4.899t_{\text{max}}^{-0.916}V_{Pi}}{365000}-\sum_{i\neq k}^{n}\frac{0.022e^{0.06T}\rho_{k}V_{Pk}p_{ik}}{W_{Bk}^{0.15}}$$

Under constant population scale,

(75) $$\frac{dV_{i}}{dt}=0$$

(76) $$0.00586\;{\left(1.113\right)}^{T-20}\;{\left(1000W_{Bi}\right)}^{-0.2}\;V_{Pi}=\frac{4.899t_{\text{max}}^{-0.916}V_{Pi}}{365000}+\sum_{i\neq k}^{n}\frac{0.022e^{0.06T}\rho_{k}V_{Pk}p_{ik}}{W_{Bk}^{0.15}}$$

If we knew the food web details and the size of primary producer, it is easy to get all the rest populations as long as they are connected to the food web. In contrast, if the population of the species is known, we can also use this formula to calculate the correct food composition of each species.

### Data validation and case study

We apply the new model to a series of case studies on polychlorinated biphenyl (PCBs) transport in Lake Ontario. The results are published in a recent article in *Environmental Pollution*: Modeling the impact of biota on polychlorinated biphenyls (PCBs) fate and transport in Lake Ontario using a population-based multi-compartment fugacity approach [20]. (https://doi.org/10.1016/j.envpol.2018.05.068).
